# The 3C’s: COVID-19, Children, and Cardiac Surgery - Do we know enough?

**DOI:** 10.21470/1678-9741-2020-0255

**Published:** 2020

**Authors:** Sudhansoo Khanna

**Affiliations:** 1 Department of Cardiovascular and Thoracic Surgery, Post Graduate Institute of Medical Education and Research (PGIMER), Chandigarh, India.

To the Editor,

Coronavirus disease 2019 (COVID-19) is major health concern that has evolved exponentially into a pandemic. The dynamics of this global health hazard are changing from moment to moment and its trajectory seems unpredictable. As of May 19, 2020, there have been 4,735,622 confirmed cases of COVID-19 with 316,289 confirmed deaths reported worldwide and the clock is still ticking^[1]^.

Coronaviruses (CoVs) are traditionally known to be non-lethal to human population, causing the ‘common cold’ in otherwise healthy people. CoVs received our due attention in 2002 and 2012 with the emergence of 2 highly pathogenic human CoVs-severe acute respiratory syndrome coronavirus (SARS-CoV) and Middle East respiratory syndrome coronavirus (MERS-CoV), respectively^[2]^. Likewise, severe acute respiratory syndrome virus 2 (SARS-CoV-2) has proved to be yet another highly pathogenic form of coronavirus, just more challenging than the previous ones. For any disease to become a pandemic, it must have a critical balance between its contagiousness and deadliness. COVID-19 manifests as a mild disease in most individuals and this, along with its highly contagious nature, has enabled COVID-19 to become a pandemic.

Although the exact clinical course, severity, and complications of COVID-19 are not yet completely determined, the risk of mortality is seen to be higher in those with age >60 years, underlying co-morbid conditions like diabetes mellitus, underlying cardiac or lung disorder^[3]^. This fact is of paramount importance for any healthcare provider dealing with cardiac sciences, more so to cardiac surgeons.

On the other hand, COVID-19 appears to sympathize with the children in general. COVID-19 affects children; however, the severity of the disease is milder, and the overall prognosis is better than adults. Furthermore, mortality is an extremely rare phenomenon in children infected with COVID-19^[4]^. Several hypotheses have been suggested but these remains to be proved. First, angiotensin-converting enzyme 2 (ACE2) has been proved to be a functional receptor for SARS-CoV-2 and children may be protected against SARS-CoV-2 as this enzyme is less mature at younger ages^[5]^. Second, ‘trained immunity’ in response to frequent viral infections in childhood may seem protective. Third, a higher constitutional lymphocyte count in children is proposed as a protection mechanism against SARS-CoV-2^[6]^. However, the actual reason may continue to be a mystery due to the smaller number of immunological studies available to date due to the smaller number of infected patients in the pediatric population.

CoVs are known to affect the cardiovascular system. However, there are significant gaps in our current understanding of the pathophysiology and effects of COVID-19, especially on the cardiovascular system. Although several mechanisms have been proposed, such as eliciting a ‘cytotoxic storm’, systemic inflammatory response syndrome (SIRS), plaque instability and even cases of direct cytotoxic effects on myocardium (myocarditis) have been reported, SIRS appears to be the most important mechanism^[7]^. Cardiopulmonary bypass (CPB) is an integral component of most corrective cardiac surgeries. CPB is known to produce SIRS, which can cause myocardial and pulmonary dysfunction in the postoperative period^[8]^. Therefore, it is easily comprehensible that a nosocomial SARS-CoV-2 infection postoperatively after cardiac surgery under CPB can be potentially lethal due to the compound impact of inflammatory response by both CPB and SARS-CoV-2.

SARS-CoV-2 has a unique marked affinity towards host ACE2 receptor. ACE2 receptor-dependent entry of SARS-CoV-2 has put forward a therapeutic dilemma. On the one hand, ACE inhibitors (ACEIs) or angiotensin receptor blockers (ARBs) can increase the viral entry into host cells by compensatory up-regulating ACE2 receptors, thus making the individuals on these drugs more susceptible to SARS-CoV-2. On the other hand, compensatory up-regulation of these ACE2 receptors may provide a protective effect against inflammatory response of SARS-CoV-2^[7]^. Currently, we have no conclusive evidence regarding the discontinuation of ACEIs/ARBs in a case of COVID-19^[9]^.

Several trials are underway in our pursuit to find a vaccine or therapeutic drug against SARS-CoV-2. Anti-malarials, like hydroxychloroquine and a couple of anti-viral drugs, have been proposed, but their efficacy is doubtful to say the least. We currently have no prophylactic vaccine or definitive curative treatment against SARS-CoV-2.

CoVs seem to have mastered the art of deception, in the way it evolves every few years to cross the species barrier, resulting into epidemics or a pandemic every 10 years for the past 3 decades. The global catastrophe created by another member of the CoV family, which was previously thought to be quite benign, has posed serious questions regarding our preparedness to deal with this ever-evolving set of zoonotic diseases. We must solve this mystery surrounding CoVs so that *history may not repeat itself once again in the future.*

Although COVID-19 emerged as an acute infectious pandemic, it could soon evolve into a chronic epidemic similar to influenza due to genetic recombination. Thus, we will repeatedly face this critical issue of conducting cardiac surgery under CPB in children in this era of COVID-19. Until we obtain conclusive data regarding the management of pediatric cardiac patients with COVID-19, we must rely on our clinical judgment.
